# Factor retention in ordered categorical variables: Benefits and costs of polychoric correlations in eigenvalue-based testing

**DOI:** 10.3758/s13428-024-02417-0

**Published:** 2024-05-06

**Authors:** Nils Brandenburg

**Affiliations:** Department of Experimental Psychology, Heinrich Heine University Düsseldorf, Universitätsstr. 1, 40225 Düsseldorf, Germany

**Keywords:** Exploratory factor analysis, Factor retention, Next Eigenvalue Sufficiency Test, Ordinal variables, Polychoric correlations

## Abstract

An essential step in exploratory factor analysis is to determine the optimal number of factors. The Next Eigenvalue Sufficiency Test (NEST; Achim, 2017) is a recent proposal to determine the number of factors based on significance tests of the statistical contributions of candidate factors indicated by eigenvalues of sample correlation matrices. Previous simulation studies have shown NEST to recover the optimal number of factors in simulated datasets with high accuracy. However, these studies have focused on continuous variables. The present work addresses the performance of NEST for ordinal data. It has been debated whether factor models – and thus also the optimal number of factors – for ordinal variables should be computed for Pearson correlation matrices, which are known to underestimate correlations for ordinal datasets, or for polychoric correlation matrices, which are known to be instable. The central research question is to what extent the problems associated with Pearson correlations and polychoric correlations deteriorate NEST for ordinal datasets. Implementations of NEST tailored to ordinal datasets by utilizing polychoric correlations are proposed. In a simulation, the proposed implementations were compared to the original implementation of NEST which computes Pearson correlations even for ordinal datasets. The simulation shows that substituting polychoric correlations for Pearson correlations improves the accuracy of NEST for binary variables and large sample sizes (*N* = 500). However, the simulation also shows that the original implementation using Pearson correlations was the most accurate implementation for Likert-type variables with four response categories when item difficulties were homogeneous.

## Introduction

Factor analysis is a common method to model correlations among a set of variables as functions of a smaller number of common factors. Historically, factor analysis has played a central role in scale development as it provides assessment to what extent sets of items collectively measure a common construct (i.e., common factors; Conway & Huffcutt, 2003; Henson & Roberts, 2006; O’Leary-Kelly & Vokurka, 1998; Ziegler & Hagemann, 2015). Technically, factor analysis is used to estimate a model of the population correlation matrix for a set of variables. For a brief introduction, let $p$ be the number of analyzed variables and $\Sigma$ be the $p\times p$ population correlation matrix and $m$ be the number of common factors in the factor model of $\Sigma$. The linear factor model of $\Sigma$ is given in the following equation:

1 $$\Sigma ={\mathrm{\Lambda \Psi \Lambda }}^{{\text{T}}}+\Theta$$

In Eq. (1), $\Lambda$ is the $p\times m$ matrix that denotes regression weights (i.e., factor loadings) of common factors on the variables, $\Psi$ is the $m\times m$ matrix of correlations among common factors, and $\Theta$ is a $p\times p$ matrix that increments ${\mathrm{\Lambda \Psi \Lambda }}^{{\text{T}}}$ by contributions of components that are dissociated from common factors in that they are unique to each variable. In the following sections, ‘common factors’ are referred to simply as factors and ‘factor loadings’ are referred to as loadings.

A special case of factor analysis is exploratory factor analysis which has been designed specifically to explore links between factors and variables when no factors can be specified on theoretical grounds (Achim, 2020; Widaman, 2018). To this end, exploratory factor analysis estimates factor models without constraining loading parameters to 0 (i.e., no association between a factor and a variable is ruled out a priori). When it cannot be specified which factors inform which variable, it is likely also unknown how many factors can be assumed to underlie the dataset to begin with. Hence, exploratory factor analysis typically includes some formal determination of the number of factors prior to the parameter estimation for the actual factor model (Fabrigar et al., 1999; Fava & Velicer, 1992; Goretzko et al., 2021). It is key to determine the optimal number of factors so that the factor model retains all factors of substantive importance while none of the identified factors is spurious (Auerswald & Moshagen, 2019; Braeken & van Assen, 2017; Fabrigar et al., 1999; Fava & Velicer, 1992; Henson & Roberts, 2006; Preacher et al., 2013; Schmitt, 2011). The problem of determining the optimal number of factors is referred to as the number-of-factors problem.

Consensus on how to approach the number-of-factors problem in empirical research has yet to be reached. In fact, numerous methods to determine the number of factors have been proposed in the past decade (Achim, 2017; Braeken & van Assen, 2017; Golino & Epskamp, 2017; Goretzko & Bühner, 2020; Green et al., 2012; Ruscio & Roche, 2012). One of the recent proposals is a method coined Next Eigenvalue Sufficiency Test (NEST; Achim, 2017). The objective of the present work is to contribute to the validation and the development of NEST. Previous simulation studies (Achim, 2017; Brandenburg & Papenberg, 2022) have shown that NEST determines the number of factors more accurately than other methods like parallel analysis (Horn, 1965) and Exploratory Graph Analysis (Golino & Epskamp, 2017; Golino et al., 2020), but so far evidence from simulations has been limited to continuous variables. Here, datasets with ordinal variables were simulated in order to test whether NEST also determines the number of factors accurately for ordinal variables. It was tested whether there is a preferred implementation of NEST for ordinal variables out of a set of candidate implementations. In the following sections, it is introduced how NEST determines the number of factors for correlations among analyzed variables. Then, it is outlined how ordinal variables challenge NEST and how its performance can be expected to depend on the computation of correlations. Subsequently, implementations of NEST tailored to ordinal variables are proposed. Finally, a simulation study is reported in which the proposed implementations and the original implementation are compared for simulated ordinal variables.

## Next Eigenvalue Sufficiency Test

In general, NEST determines the number of factors in a dataset through examination of the eigenvalues of the sample correlation matrix. Eigenvalues of sample correlation matrices are also central to other methods to determine the number of factors, such as the eigenvalue-greater-than-1 rule (Guttman, 1954), the scree test (Cattell, 1966), and parallel analysis (Horn, 1965). To understand the importance of eigenvalues in the context of factor analysis, consider the term ${\mathrm{\Lambda \Psi \Lambda }}^{{\text{T}}}$ from Eq. (1). This term accounts for all common-factor related parameters; it constitutes a $p\times p$ matrix that lists model-implied pairwise correlation coefficients as off-diagonal elements and nonunique variances in variables on the main diagonal. The model-implied nonunique variance in a variable is commonly referred to as the variable’s communality. Crucially, the *k*^th^ largest eigenvalue obtained from eigenvalue-decomposition of ${\mathrm{\Lambda \Psi \Lambda }}^{{\text{T}}}$ is equal to the increment of communality across all variables that would be observed with retention of the *k*^th^ factor. Individual eigenvalues do not necessarily indicate the variance that is explained across all variables by individual factors in the final factor model since factor rotation (see Fabrigar et al., 1999) changes the degree to which latent dimensions are associated with manifest variables, thereby altering the regression weights of each dimension that would be observed without rotation. Rotation does not change the total amount of variance explained by all factors together, hence the sum of *k*^th^ largest eigenvalues of ${\mathrm{\Lambda \Psi \Lambda }}^{{\text{T}}}$ indicates the sum of variance explained by all *k* factors. Individual eigenvalues indicate thus the amount of variance that each factor contributes to the total amount of variance explained factor model, which provides useful information concerning the number of factors to be retained.

Of course, in empirical applications, there is no true model ${\mathrm{\Lambda \Psi \Lambda }}^{{\text{T}}}$ that could be examined prior to factor analysis. Therefore, eigenvalue-based methods like NEST are commonly based on the eigenvalues of sample correlation matrices. Unlike the eigenvalues of ${\mathrm{\Lambda \Psi \Lambda }}^{{\text{T}}}$, eigenvalues of a sample correlation matrix do not indicate the exact amount of variance that factors contribute to a factor model. The reason is that a sample correlation matrix accounts for some amount of unique variance (indicated by the main diagonal) whereas ${\mathrm{\Lambda \Psi \Lambda }}^{{\text{T}}}$ only contains common variance. Still, eigenvalues of sample correlation matrices provide a useful approximation of variance explained by factors, especially when there is little unique variance present in the data. The widely accepted reasoning of methods to determine the number of factors in factor analysis based on eigenvalues of sample correlation matrices can be summarized as follows: If the data-generating process of $p$ observed variables consists of $m$ distinct constructs (i.e., common factors informing multiple variables or singleton variables without common variance)**,** the sample correlation matrix has $m$ large eigenvalues that are significantly larger than the $p-m$ remaining eigenvalues. When there are no common factors and all $p$ variables are independent of each other, the $p$ eigenvalues are expected equally large and differ only due to random correlations in the sample. What separates eigenvalue-based methods from each other is how they distinguish eigenvalues that indicate the presence of a factor from eigenvalues that do not.

NEST is an iterative testing procedure of the null hypothesis that *k* factors are sufficient to estimate a factor model that fits the analyzed dataset, starting with *k* = 0. The relevant test statistic that indicates the presence of at least *k* + 1 factors – as an alternative to the null hypothesis of *k*-factor sufficiency – is the eigenvalue from the sample correlation matrix at index *k* + 1, with index 1 pointing at the largest of all sample eigenvalues ordered by descending magnitude. To test the null hypothesis, NEST first computes a reference model with *k* factors for the sample correlation matrix. Computation of adequate factor models for reference in NEST is not straightforward since factor models for sample correlation matrices require a method to separate common variance from unique variance. This separation is sometimes referred to as reduction of the sample correlation matrix (see Achim, 2017). Achim (2017) compared several reduction approaches in NEST through simulation and concluded that the preferred approach in NEST is iterative reduction until convergence on one solution for the separation of common and unique variance.

With the *k*-factor reference model computed, NEST then simulates *j* surrogate datasets under this *k*-factor model^1^ with the same sample size and number of variables as the dataset in question. For *k* = 0, surrogate datasets are sampled from a population of independent variables. For all of the *j* simulated surrogate dataset, a sample correlation matrix and its respective eigenvalues are computed. The eigenvalues at index *k* + 1 from these synthetically created sample correlation matrices thus form a sampling distribution of eigenvalues under the null hypothesis: This sampling distribution describes how the tested eigenvalue can be expected to look like if there are, in fact, no more factors than the *k* factors that have already been identified given that the surrogate datasets were simulated under the *k*-factor reference model.

NEST makes no distributional assumptions concerning the distribution of eigenvalues. Instead, NEST evaluates evidence against the null hypothesis by ranking the tested eigenvalue and all of the *j* eigenvalues from its simulated sampling distribution ordered by descending magnitude (i.e., the greatest eigenvalue is assigned rank 1, ranging from 1 to *j* + 1 with decreasing magnitude). For a given $\mathrm{\alpha }$ level, the null hypothesis is rejected if the rank of the tested eigenvalue is less than $\mathrm{\alpha }(j+1)$, indicating that the tested eigenvalue exceeds its sampling distribution under the null hypothesis given that lower ranks correspond to larger eigenvalues. If the tested eigenvalue exceeds the simulated sampling distribution, it is considered evidence for the presence of an additional factor besides the *k* factors from the* k*-factor reference model. Then, *k* is incremented by 1 and the next eigenvalue is tested with surrogate datasets under a new *k*-factor model that accounts for factor that has been confirmed in the previous step. If the tested eigenvalue does not exceed the simulated sampling distribution, NEST stops and returns *k* as the suggested number of factors.

By updating the sampling distribution of tested eigenvalues conditional on the factors for which retention has already been confirmed, NEST differs from the well-studied parallel analysis (PA), which also ranks eigenvalues of the analyzed dataset among eigenvalues of surrogate datasets. The main difference between NEST and PA is that PA samples all surrogate datasets from a reference model with independent variables. The latter has been criticized based on the argument that retaining at least one factor implies that the analyzed variables share more common variance than implied by the surrogate datasets in PA (Braeken & van Assen, 2017; Green et al., 2012; Ruscio & Roche, 2012; Saccenti & Timmerman, 2017; Turner, 1998). NEST is based on work by Green et al. (2012) who have proposed a ‘revised parallel analysis’ (RPA) with which sequential conditioning of simulated sampling distributions on every retained factor was introduced. The differences in design between NEST and RPA mainly concern the computation of the *k*-factor models to simulate surrogate datasets (for a detailed contrast between NEST and RPA, see Achim, 2017).

Previous simulation studies showed that NEST is as accurate as, or more accurate than, several PA variants for simulated datasets under a wide range of factor models (Achim, 2017; Brandenburg & Papenberg, 2022). However, in these studies NEST was tested only with continuous variables sampled from multivariate normal distributions. As will be derived in the following sections, the performance of NEST for ordinal variables instead of continuous variables requires dedicated research. The main problem at hand is that the computation of sample correlation matrices, which necessarily occurs in several steps of NEST (see above), is not as straightforward for ordinal datasets than it is for continuous datasets.

## Problems with ordinal variables

A widely regarded problem with ordinal variables is that sample product-moment correlations – hereafter referred to as Pearson correlations – underestimate the true correlations among ordinal variables if these ordinal variables are mere discretized representations of latent continuous variables (not to be confused with latent factors; Garrido et al., 2013; Green et al., 2016; Lubbe, 2019).

Building on the idea of ordinal variables as discretized variants of latent continuous variables, sample *polychoric correlations* do not compute the correlation among observed ordinal variables directly but the correlation among the assumed latent continuous variables underlying the ordinal variables (Flora & Curran, 2004; Jin & Yang-Wallentin, 2017; Muthén, 1978; Olsson, 1979a). Assuming that the latent variables are normally distributed, it can be shown that maximum-likelihood estimation of polychoric correlations is asymptotically unbiased, which is an advantage over biased Pearson correlations (Lubbe, 2019). However, even if distributional assumptions are met, computing polychoric correlations instead of Pearson correlations comes at the cost of an increased standard error (i.e., instable sample correlations in repeated sampling from the population; Garrido et al., 2013, 2016; Roznowski et al., 1991; Tran & Formann, 2009; Weng & Cheng, 2017). Concerning factor analysis for ordinal variables, it has been debated whether there are substantial benefits to the estimation of factor models for polychoric correlation matrices instead of Pearson correlation matrices (Flora & Curran, 2004; Garrido et al., 2016; Goretzko et al., 2021). In the present work, the focus is not on the general use of polychoric correlations in factor analysis but on the use of polychoric correlations specifically to determine the number of factors through eigenvalues of sample correlation matrices. It was assumed that there is an optimal number of factors on population-level and the aim was to investigate whether this number is more easily recovered in sample correlation matrices with Pearson correlations or polychoric correlations. No guidance is provided here regarding the choice of correlation type in the subsequent parameter estimation for a factor model following the recovery of the number of factors (see Flora & Curran, 2004, for a more in-depth discussion on polychoric correlations in factor analysis for ordinal data).

The original implementation of NEST, published by Achim (2017), had been developed to test NEST for continuous variables using Pearson correlations. In the original implementation continuous surrogate datasets are simulated from a multivariate normal distribution implied by the tested *k*-factor model of Pearson correlations in the observed data. Pearson correlations are also computed for the simulated surrogate datasets. Central research questions in the present work concern how accurately NEST determines the optimal number of factors for ordinal datasets when Pearson correlations are computed and whether accuracy improves when polychoric correlations are computed instead. The following sections explain different proposals at which steps in NEST the computation of Pearson correlations can be changed to polychoric correlations. For the moment, imagine that wherever the original implementation computes Pearson correlation matrices (i.e., for the analyzed sample and all simulated surrogate samples at every iteration of *k*), polychoric correlation matrices are computed instead. The well-known bias of Pearson correlations on the one hand and the inflated standard error of polychoric correlations on the other suggest that one should be careful before adopting a preference for one approach over the other. Similar work in which PA with Pearson correlations was contrasted to PA with polychoric correlations has yielded mixed results (Cho et al., 2009; Garrido et al., 2013; Lubbe, 2019; Timmerman & Lorenzo-Seva, 2011; Tran & Formann, 2009; Weng & Cheng, 2005).

The differences in eigenvalues from Pearson correlation matrices and polychoric correlation matrices have implications for NEST. Simulations by Lubbe (2019) have shown these differences remarkably clearly. First, biased Pearson correlations for ordinal variables result in lower signal eigenvalues (i.e., eigenvalues corresponding to factors that should be retained) compared to unbiased Pearson correlations for continuous variables. This is relevant to NEST because a factor corresponding to a large signal eigenvalue is more likely to be retained than a factor corresponding to a small signal eigenvalue. Simply put: the greater the smallest signal eigenvalue to be detected, the greater the statistical power of NEST. Second, the increased standard error of polychoric correlations increases the variance of signal eigenvalues and noise eigenvalues (i.e., eigenvalues not corresponding to factors) compared to Pearson correlations for ordinal variables. This, in turn, is relevant to NEST because greater dispersion of signal eigenvalues implies that the smallest signal eigenvalue is lower and therefore less likely to be retained by NEST than with Pearson correlations for continuous variables. In conclusion, the danger of reduced statistical power that can be derived with Pearson correlations also occurs with polychoric correlations, albeit for reasons linked to standard error rather than bias.

What is more, Lubbe (2019) has shown that both the bias of Pearson correlations and the standard errors of polychoric correlations increase for ordinal variables when category probabilities within variables – which correspond to item difficulties – are asymmetric and increase even further when category probabilities vary among variables in a dataset. In contrast, the standard error of Pearson correlations and the unbiasedness of polychoric correlations were mostly insensitive to manipulated category probabilities.

From these observations it can be anticipated that NEST is generally more likely to underestimate the number of factors for ordinal variables due to decremented statistical power compared to applications of NEST with Pearson correlations for continuous datasets. Decremented power for ordinal variables can be anticipated with Pearson correlations due to bias and with polychoric correlations due to standard error. The investigations reported here also took varying category probabilities in ordinal datasets into account. This was done due to the effects on eigenvalues reported by Lubbe (2019) from which it may be inferred that the performance of NEST for ordinal datasets may depend not only on the computation of correlations but also on the interaction between the computation of correlations and category probabilities.

Additionally, in instances where NEST has identified every signal dimension, varying category probabilities in a dataset also may promote overestimation of the number of factors. This potential problem can be anticipated to affect NEST when Pearson correlations are computed for ordinal variables and continuous surrogate datasets as it is done in the original implementation of NEST (Achim, 2017). Recalling the aforementioned idea of ordinal variables as discretized variants of continuous constructs, Pearson correlations between discretized values that are similar in the continuous domain are sensitive to cut-off values in the continuous domain which govern the category probabilities in the discrete domain. When two ordinal indicators of the same common factor (i.e., two correlated ordinal variables) have different sets of category probabilities due to different cut-off values in their respective continuous representations, pairs that are similar in value in the continuous domain may fall into different response categories. This would affect the observed Pearson correlation between the pair of ordinal variables in a manner that is not related to the common factor (Garrido et al., 2013; Green et al., 2016; Lim & Jahng, 2019; Olsson, 1979b; Tran & Formann, 2009; Yang & Xia, 2015). In consequence, in a sample correlation matrix of variables with heterogeneous category probabilities, other eigenvalues besides to the ones associated with whatever factors inform the variables can be observed elevated above the rest of the eigenvalues to account for the additional determinant of correlations (Garrido et al., 2013; Lim & Jahng, 2019). These additional determinants based on category probabilities (i.e., item difficulties) are hence sometimes referred to as *difficulty factors*.^2^ These are not to be confused with the common factors whose number is to be determined in the number-of-factors problem since difficulty factors are not viewed as distinct constructs measured through indicator variables but as mere results of item difficulties (Lim & Jahng, 2019; Lubbe, 2019; Tran & Formann, 2009).

In the following simulation-based assessment of methods to determine the number of factors, it is therefore assumed that difficulty factors are spurious (Cho et al., 2009; Olsson, 1979b; Yang & Xia, 2015) and retention of difficulty factors is considered erroneous with respect to the optimal number of factors (parallel to Garrido et al., 2013; Lim & Jahng, 2019; Tran & Formann, 2009). This assumption will be revisited in the discussion below.

Returning to the issue of NEST, given that continuous surrogate datasets in the original implementation of NEST do not account for the presence of difficulty factors in the analyzed dataset, tested eigenvalues corresponding to difficulty factors may exceed their simulated sampling distribution. This would likely cause NEST to reject the null hypothesis of *k*-factor sufficiency erroneously due to ill-behaved simulations of sampling distributions for tested eigenvalues.

## Simulation of ordinal variables

To investigate whether Pearson correlations or polychoric correlations result in more accurate performance by NEST for ordinal variables, a simulation method for ordinal variables under factor models designed by Yang and Xia (2015) was adopted. Specifically, their simulation method was adopted as it includes instructions to manipulate category probabilities. The three levels of category probabilities in the design by Yang and Xia can be described as symmetric, invariant asymmetric, and varying asymmetric. These levels distinctly impact the bias of Pearson correlations and the standard errors of polychoric correlations according to Lubbe (2019) and account for difficulty factors through varying asymmetric category probabilities.

The method by Yang and Xia (2015) starts by sampling a continuous dataset from a multivariate normal distribution with a covariance matrix that is determined by a factor model. At the population-level, the marginal distribution of each variable is a standard normal distribution. Each normal value is then transformed into an ordinal value depending on whether the normal value exceeds a predetermined threshold corresponding to intervals under the normal distribution curve, yielding specific category probabilities. Using threshold values in accordance to the normal distribution is common practice in simulations of ordinal variables (Cho et al., 2009; Garrido et al., 2013; Green et al., 2016). Yang and Xia reported separate thresholds that transform normal variables into ordinal variables with two response categories (i.e., binary variables) and variables with four response categories. The thresholds for symmetric category probabilities were {0.00} for two categories and {– 1.00, 0.00, 1.00} for four categories. For invariant asymmetric category probabilities, the thresholds were {1.00} for two categories and {0.00, 0.75, 1.50} for four categories. The invariant asymmetric thresholds were used for all variables in a dataset, resulting in equally skewed category probability distributions in all variables. For varying asymmetric category probabilities, the same thresholds from the invariant asymmetric condition were used, albeit in sign-reversed form for every second variable. Following this method, symmetric category probabilities and invariant asymmetric category probabilities imply homogeneous item difficulties while varying asymmetric category probabilities imply heterogeneous item difficulties.

**Fig. 1:**
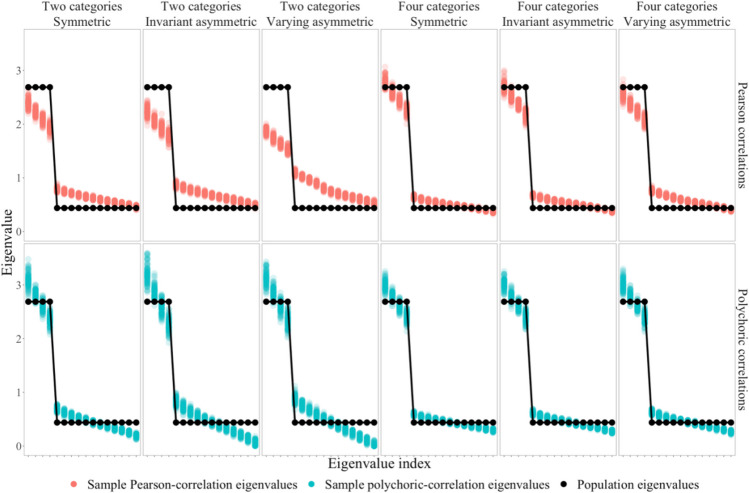
Sample eigenvalues as a function of correlation measure, response categories, and category probabilities. *Note*. The datasets in this figure were simulated under a factor model with four orthogonal factors, indicated by four variables each with loadings of 0.75 and without cross-loadings. Population eigenvalues are the eigenvalues of the model-implied correlation matrix. The sample size was set to *N* = 500 in all simulated datasets. All cells summarize an independent set of datasets, accounting for the respective correlation measure, number of response categories, and category probabilities. A total of 100 datasets were sampled per cell

The sections above already established that Pearson correlations for ordinal datasets simulated in such fashion suffer from bias while polychoric correlations suffer from increased standard error. To illustrate how the method by Yang and Xia (2015) affects eigenvalues of Pearson correlation matrices and polychoric correlation matrices respectively, datasets with two response categories and datasets with four response categories were simulated under a factor model. The eigenvalues of the sample correlation matrices were then compared to the eigenvalues of the model-implied correlation matrix as population-level reference in Fig. 1. Additional technical details of this simulation – and of the simulations reported in the following sections – are explained in Appendix A. With Pearson correlations, underestimations of population-level correlations in samples caused signal eigenvalues to be lower than their population-level reference. Conversely, noise eigenvalues of Pearson correlation matrices exceeded population-level noise eigenvalues. With polychoric correlations,^3^ the eigenvalues in Fig. 1 reflect the increased standard error in two aspects. First, the dispersion of signal eigenvalues within each index was greater compared to Pearson correlations. Second, the dispersion of all signal eigenvalues and all noise eigenvalues around their population-level reference was greater than with Pearson correlations. Figure 1 also shows that the effects of bias and standard error on sample eigenvalues were stronger (a) for asymmetric category probabilities (both invariant and varying) than for symmetric probabilities and (b) for binary variables than for variables with four response categories.

Of course, Fig. 1 has mainly illustrative purposes and does not allow firm conclusions about whether Pearson correlations or polychoric correlations are superior in NEST for ordinal variables. Such firm conclusions may be derived from large-scale simulations in which different implementations of NEST with both types of correlations are applied to simulated datasets under an array of conditions.

## NEST with polychoric correlations

In this section, two candidate implementations of NEST making use of polychoric correlations are proposed. There are two possible approaches to counteract expected problems with the original NEST implementation for ordinal datasets. The first one that comes to mind is to compute a sample correlation matrix of polychoric correlations instead of Pearson correlations and to test its eigenvalues in turn. Implementing a NEST variant that tests eigenvalues from polychoric correlation matrices is not trivial due to the requirement to simulate sampling distributions of eigenvalues under the null hypothesis of *k*-factor sufficiency. This sampling distribution must be based also on polychoric correlations to accommodate the polychoric correlations from the analyzed dataset. Figure 1 shows that eigenvalues from datasets under factor models are sensitive to the standard errors of sample correlations. Hence, computing polychoric correlations in a dataset, then computing a *k*-factor reference model from this sample polychoric correlation matrix, and then adhering the original implementation by simulating continuous surrogate datasets and computing Pearson correlations for the surrogate datasets would simulate sampling distributions of tested eigenvalues that are less dispersed than the actual sampling distributions of eigenvalues of polychoric correlation matrices. Consequently, using polychoric correlations for a dataset and Pearson correlations for continuous surrogate datasets in NEST would frequently suggest rejecting the null hypothesis when the null hypothesis is actually true. This is so because the largest noise eigenvalues of polychoric correlation matrices can be expected to exceed the largest noise eigenvalues of Pearson correlation matrices due to the increased standard error of polychoric correlations (see Fig. 1).^4^

To provide adequate sampling distributions of eigenvalues of polychoric correlation matrices, the first proposed implementation of NEST built on polychoric correlations computes polychoric correlations for the analyzed dataset as well as the surrogate datasets. Computation of polychoric correlations for surrogate datasets requires simulation of ordinal variables in these surrogate datasets. Furthermore, to achieve compatible standard errors of polychoric correlations between the analyzed dataset and its surrogate datasets, ordinal surrogate datasets need to be simulated with category probabilities that match the probabilities of the corresponding variables from the analyzed dataset (Lubbe, 2019). In the proposed implementation of NEST, the simulation of ordinal surrogate datasets was done similar to the aforementioned simulation routine for ordinal datasets by Lim and Jahng (2015): In a first step, category probabilities are calculated for each ordinal variable in the analyzed dataset. Then, continuous surrogate datasets are simulated under the multivariate normal distribution implied by the *k*-factor model of the analyzed sample correlation matrix. Next, for each variable in each surrogate dataset, the quantiles of the variables’ simulated values are computed according to the category probabilities observed in the corresponding variable. Quantiles are used as thresholds to transform continuous surrogate datasets into ordinal datasets. This approach has been suggested by A. Achim – the author of the original implementation of NEST (personal communication, June 28, 2021). Specifying thresholds for each variable in each surrogate dataset separately using its individual quantiles guarantees that all variables in surrogate datasets include the same number of response categories as the variables from which the surrogate datasets were derived. Otherwise, computation of polychoric correlations may fail in surrogate datasets due to inconsistent numbers of categories. Appendix A includes technical details of the handling of the computational costs of computing polychoric correlations for each surrogate dataset and the handling of nondefinite correlation matrices.

In summary, the first proposed implementation of NEST tailored to ordinal variables first computes a polychoric sample correlation matrix. The *k*-factor reference models are then computed from this polychoric sample correlation matrix. Surrogate datasets are then artificially discretized with respect to the observed category probabilities in each variable. Finally, polychoric correlations are computed for each surrogate datasets in order to provide an adequate sampling distribution for the tested eigenvalues which also stem from polychoric correlations. Mind that this proposal should not be considered an improvement over the original implementation without thorough simulation-based investigation due to the concerns related to the large standard error of polychoric correlations. It is therefore worthwhile to consider also another possible implementation of NEST that tackles the issues of the original implementation for ordinal datasets.

The second proposed NEST implementation was also originally suggested by the original author of NEST (A. Achim, personal communication, June 28, 2021). It is mainly targeted at the issue that the original implementation fails to account for difficulty factors and can thus be expected to overestimate the number of factors in their presence. Remember that this problem can be expected to occur whenever the sample Pearson correlation matrix for an analyzed dataset with ordinal variables is influenced by difficulty factors but the Pearson correlation matrices for continuous surrogate datasets are not (see above). The second proposed implementation hinges on the idea that failure to account for difficulty factors may be solved even when sticking to Pearson correlations through the simulation of ordinal surrogate datasets instead of continuous surrogate datasets. When ordinal variables in surrogate datasets replicate the observed category probabilities from the corresponding variables from the analyzed dataset – just as explained above for adequate sampling distributions in the first proposal –, the difficulty factors that affect the analyzed sample Pearson correlations also affect Pearson correlations for the surrogate datasets. Therefore, applying the same routine to reproduce category probabilities in surrogate datasets that was suggested in the previous sections can be expected to prevent NEST from overestimating the number of factors in the presence of difficulty factors even when Pearson correlations are used to obtain eigenvalues.

While the second proposed implementation of NEST continues to test eigenvalues from Pearson correlation matrices for ordinal variables, adequate surrogate datasets can only be achieved when the *k*-factor reference models are computed for a polychoric sample correlation matrix of the analyzed dataset. The reason for this lies in the underestimation of Pearson correlations for ordinal variables. If a reference model were to be computed for underestimated Pearson correlations, ordinal surrogate datasets under the model would not reproduce the model-implied correlations. Instead, Pearson correlations for the ordinal surrogate datasets would again underestimate population-level correlations (i.e., the correlations implied by *k*-factor reference model), thereby misrepresenting the amount of variance explained by the *k* factors which is meant to be reproduced in the surrogate datasets, thus causing all eigenvalues from surrogate datasets to be distributed differently than the tested eigenvalues. Repeated underestimation is not a problem for the original NEST implementation which uses continuous surrogate datasets for which Pearson correlations are unbiased. Using polychoric correlations to compute a *k*-factor reference model allows the eigenvalues corresponding to the first *k* factors for the surrogate datasets under the model to align with the eigenvalues from the Pearson correlation matrix for the analyzed dataset: When Pearson correlations for an analyzed ordinal dataset underestimate population-level correlations to some extent, polychoric correlations provide an unbiased estimate of the same population-level correlations that can be used to build a model for the simulation of ordinal surrogate datasets. Then, Pearson correlations for the ordinal surrogate datasets under the model of the polychoric correlations underestimate the population-level correlations to the same extent as in the analyzed dataset.

In summary, the second proposed implementation of NEST tests eigenvalues from Pearson correlations when applied to ordinal datasets. Sampling distributions for the tested eigenvalues are provided by first computing an auxiliary polychoric correlation matrix of the same analyzed dataset and then simulating ordinal surrogate datasets – which reproduce observed category probabilities – under reference models of the polychoric correlation matrix. Again, without extensive testing simulations, it is yet unknown how the second proposal compares to the original NEST implementation and the prior proposal that exclusively relies on polychoric correlations. While the replication of observed category probabilities promises protection against overestimation due to difficulty factors in contrast to the original implementation, the problem of decremented power due to reduced signal eigenvalues from Pearson correlations remains. What is more, the mix of Pearson correlations and polychoric correlations in the second proposal make it difficult to predict if the simulated sampling distributions for signal eigenvalues provide greater power than in the other two NEST variants and to what extent the second proposal suffers from both the issues related to Pearson correlations and polychoric correlations.

## Simulation study

A simulation study was conducted to compare the performance of the original implementation of NEST when applied to ordinal datasets to the two proposed alternative implementations. The goal was to test (a) whether the anticipated problems could indeed be observed for ordinal datasets, (b) how severely the performance of the variants of NEST would deteriorate, and (c) how the three variants performed in direct comparison to investigate whether polychoric correlations offer benefits to the performance of NEST for ordinal variables.

### Simulated data structures

In the present simulation study, seven independent variables were manipulated in a fully crossed design: the true number of factors (2, 4), the number of variables per factor (4, 7), the distribution of loading parameters (𝒰(0.40, 0.50), 𝒰(0.70, 0.80)), the inter-factor correlation parameters (0.20, 0.70), the sample size (*N*) of datasets (100, 500), the number of response categories (two categories, four categories), and category probabilities in variables (symmetric, invariant asymmetric, varying asymmetric). Combined, the design implied 192 conditions. For each condition 100 datasets were simulated, resulting in 19.200 simulated datasets in total.

Each condition implied a family of factor models according to Eq. (1). The number of variables was the product of the number of factors and the number of variables per factor. Each factor was indicated through nonzero loadings by the number of variables per factor. Each nonzero loading parameter was independently sampled from the according uniform distribution to simulate heterogeneity of loadings on population level. Consequently, each variable only had one nonzero loading parameter, implying perfect simple-structure models (Revelle & Rocklin, 1979). All off-diagonal elements of the inter-factor correlation matrix were set to the inter-factor correlation parameter according to the simulation’s design (see above). Together, these manipulations implied the term ${\mathrm{\Lambda \Psi \Lambda }}^{{\text{T}}}$ from Eq. (1), which was transformed into the model-implied correlation matrix by incrementing its main-diagonal elements to 1. It follows that only common factors determined the population correlation matrix. There was no source of correlation at the population level other than that implied by ${\mathrm{\Lambda \Psi \Lambda }}^{{\text{T}}}$. Given that the manipulation of the loading parameters involved random number sampling, 100 factor models per condition were generated and one dataset per factor model was simulated to achieve 100 datasets per condition (parallel to Brandenburg & Papenberg, 2022). Response categories and category probabilities were manipulated as suggested by Yang and Xia (2015). The levels of response categories and category probabilities in the simulation study were the same as those illustrated in Fig. 1. All simulated variables were originally sampled from a multivariate normal distribution implied by the respective factor model and were transformed into ordinal datasets according to the method by Yang and Xia.

Due to the computational costs of polychoric correlations (which mainly affected the first proposed implementation of NEST given its use of polychoric correlations for surrogate datasets), the present simulation study was designed with a smaller range of conditions than previous simulations that had applied NEST to continuous variables (Brandenburg & Papenberg, 2022). The levels of the independent variables that were unrelated to the categorization method by Yang and Xia (2015) were specified with the intention to avoid bottom and ceiling effects in the performance of NEST variants. Specifically, the statistical power of NEST diminishes (a) as the number of factors and the inter-factor correlations increase, and (b) as the number of variables per factor, loading parameters, and sample size decrease (Auerswald & Moshagen, 2019; Braeken & van Assen, 2017; Brandenburg & Papenberg, 2022; Lim & Jahng, 2019; Lubbe, 2019). Hence, two levels were selected for each of these independent variables to include an ‘easy’ and a’difficult’ level combined in a fully crossed design.

### Investigated methods

The three competing NEST variants were applied to all 19,200 simulated datasets to investigate how accurately they recovered the number of factors of the factor models under which the datasets had been simulated. In the following sections, the original implementation that relies entirely on Pearson correlations is referred to as NEST_Pearson_, the first proposed implementation that relies entirely on polychoric correlations is referred to as NEST_poly_, and the second proposal that combined Pearson correlations for eigenvalue testing and polychoric correlations for data simulation is referred to as NEST_hybrid_. For all three variants the null hypothesis of *k*-factor sufficiency was tested with 200 surrogate datasets for each test and $\mathrm{\alpha }$ = 0.05. The computation of *k*-factor models from which to simulate surrogate datasets was done through iterative reduction of sample correlation matrices in all NEST implementations in accordance to Achim (2017).

Additionally, a variant of PA was applied to all simulated datasets to provide a benchmark for the performance of NEST. PA is frequently used to add some benchmark in comparative simulations (Achim, 2017; Braeken & van Assen, 2017; Golino et al., 2020; Goretzko & Bühner, 2020; Lorenzo-Seva et al., 2011; Ruscio & Roche, 2012). Here the implementation of PA published by Lubbe (2019) was adopted. Lubbe’s implementation is tailored specifically to ordinal datasets: Polychoric correlations are computed for the analyzed dataset and surrogate datasets while all variables in surrogate datasets reproduce the observed category probabilities (as in the proposed NEST_poly_ implementation). This PA implementation involves no reduction of sample correlation matrices (see Auerswald & Moshagen, 2019). Lubbe conducted a simulation study and concluded that polychoric correlations and reproduced category probabilities in surrogate datasets in PA are key to optimal performance for ordinal datasets. Therefore, their implementation was used in the present simulation to explore how it compares to NEST_Pearson_, NEST_poly_, and NEST_hybrid_. To highlight that this implementation of PA computes polychoric correlations in every step, it is referred to as PA_poly_. The software solutions to compute polychoric correlations were the same in NEST_poly_, NEST_hybrid_, and PA_poly_ (see Appendix A). The number of surrogate datasets in PA_poly_ was set to 200 for consistency with the NEST variants. As suggested by Lubbe, the threshold of reference eigenvalues which the tested eigenvalues had to exceed in order to retain the corresponding factor was the 50th percentile.

### Analysis

The number of factors suggested by NEST_Pearson_, NEST_poly_, NEST_hybrid_, and PA_poly_ was recorded for all simulated datasets. As in a previous simulation study on NEST (Brandenburg & Papenberg, 2022), each solution was labeled according to one of four (exhaustive) outcomes: a solution was ‘accurate’ if the number of recovered factors was equal to the ground-truth number of factors form the factor model under which the analyzed dataset had been simulated. The accuracy of a method was defined as the proportion of its accurate solutions in all four possible outcomes. A solution was labeled ‘overestimated’ if the number of recovered factors exceeded the ground-truth number of factors and ‘underestimated’ if the number of recovered factors was lower than the ground-truth number of factors. An overestimated solution can be regarded a type 1 error (i.e., the null hypothesis is rejected while testing a noise eigenvalue) and an underestimated solution can be regarded a type 2 error (i.e., the null hypothesis was not rejected while testing a signal eigenvalue). Finally, a solution was ‘undefined’ if the implementation failed to return any solution.

### Pilot simulation

The power of NEST_Pearson_, NEST_poly_, NEST_hybrid_ was anticipated to be decremented for ordinal datasets compared to NEST_Pearson_ for continuous datasets. Also, the potential presence of difficulty factors in Pearson correlation matrices for ordinal datasets was anticipated to increase the risk of overestimation by NEST_Pearson_ compared to applications of NEST_Pearson_ for continuous datasets. Therefore, a pilot simulation was conducted in which NEST_Pearson_ was applied to continuous datasets in order to obtain a baseline performance of NEST_Pearson_. The pilot simulation included the same manipulations of the number of factors, the number of variables per factor, loading parameters, inter-factor correlation parameters, and sample size as the design introduced above. Consistent with main simulation, 100 datasets per condition were sampled from the model-implied multivariate normal distributions. The marginal probability distribution of all variables was approximately symmetric.

### Availability

The source code of all reported simulations can be retrieved from the Open Science Repository (see https://osf.io/wb2ys/) that accompanies this manuscript. This repository also contains the raw data from the present simulation study, scripts to replicate the analyses of the raw data, and the implementations of NEST_Pearson_, NEST_poly_, NEST_hybrid_, and PA_poly_. Furthermore, the repository includes instructions on how to replicate the present simulation, either by re-simulating the same datasets that had been simulated in the present work or by simulating new datasets under the same conditions. The implementation of the present simulation can be adjusted to account for different sets of conditions. Also, instructions are provided to run simulations with different methods to determine the number of factors than those discussed here.

### Results

The simulation indicated that the performance of NEST_Pearson_, NEST_poly_, NEST_hybrid_, and PA_poly_ were sensitive to the number of response categories, the level of category probabilities, and sample size (indicated by their respective accuracy listed in the following tables). These effects are particularly interesting for empirical research as these conditions were unrelated to factor models. Hence, in practice, these conditions can be assessed prior to applications of NEST. Performance is reported separately for the numbers of response categories, the levels of category probabilities, and sample sizes.

#### Two response categories

Table 1 lists the proportions of outcomes for binary datasets depending on sample size and category probabilities. NEST_poly_ was the most accurate method for binary datasets with *N* = 500 averaged across all category probabilities (67.2% accurate, 30.5% underestimation, 2.3% overestimation). With *N* = 100, PA_poly_ was the most accurate method averaged across all category probabilities (36.1% accurate, 53.6% underestimation, 10.3% overestimation) while NEST_poly_ was the least accurate method (28.3% accurate, 70.8% underestimation, 0.7% overestimation, 0.3% undefined).

**Table 1:** Outcomes for binary datasets as a function of sample size and category probabilities

Method	Outcome	*N* = 100			*N* = 500		
		Symmetric	Invariant asymmetric	Varying asymmetric	Symmetric	Invariant asymmetric	Varying asymmetric
NEST_Pearson_	Overestimation	2.8	16.1	11.5	3.7	17.1	44.1
	Accurate	42.6	32.5	18.4	75.9	58.9	21.1
	Underestimation	54.6	51.4	70.1	20.4	24.0	34.8
	Undefined	0	0	0	0	0	0
NEST_poly_	Overestimation	1.0	0.5	0.6	2.2	1.6	3.0
	Accurate	39.7	26.4	18.8	77.1	64.7	59.9
	Underestimation	59.3	72.3	80.7	20.6	33.7	37.1
	Undefined	0	0.8	0	0	0	0
NEST_hybrid_	Overestimation	1.0	1.2	3.2	2.3	2.7	2.0
	Accurate	41.9	32.4	18.2	77.2	68.3	41.9
	Underestimation	57.1	66.4	78.5	20.5	29.0	56.1
	Undefined	0	0	0	0	0	0
PA_poly_	Overestimation	9.4	12.1	9.4	2.2	7.7	8.9
	Accurate	41.9	36.4	30.0	63.5	52.1	49.7
	Underestimation	48.6	51.5	60.6	34.3	40.2	41.4
	Undefined	0	0	0	0	0	0

Percentage of outcomes in all simulated binary datasets.

In general, all methods performed best with *N* = 500 and symmetric category probabilities. As for the sample size, all methods underestimated the number of factors less frequently with *N* = 500 than with *N* = 100. The decreased type 2 error rate with increased sample size illustrates how the power of NEST increases with sample size. With respect to category probabilities, all methods were most accurate with symmetric category probabilities, less accurate with invariant asymmetric category probabilities, and least accurate with varying asymmetric category probabilities.

Table 1 indicates that reduced accuracy with the asymmetric category probability levels in NEST_poly_ and NEST_hybrid_ can be attributed to underestimations and not to overestimations. NEST_poly_ and NEST_hybrid_ rarely overestimated the number of factors and did not exceed the normative type 1 error rate of 5% (implied by their $\mathrm{\alpha }$ level). In contrast, asymmetric category probabilities simultaneously caused more underestimations and overestimations by NEST_Pearson_. The only exception was that NEST_Pearson_ showed less underestimations with invariant asymmetric category probabilities than with symmetric probabilities with *N* = 100. Overall, this indicates that NEST_Pearson_ likely suffered from reduced power as well as sensitivity to difficulty factors. A striking problem with NEST_Pearson_ was that it overestimated the number of factors more frequently with *N* = 500 than with *N* = 100. Table 1 shows that overestimations by NEST_Pearson_ were particularly frequent with *N* = 500 and varying asymmetric category probabilities.

In comparison, NEST_poly_ underestimated the number of factors more frequently than NEST_Pearson_ with all sample sizes and category probabilities. Hence, the power of NEST_poly_ was considerably lower than the power of NEST_Pearson_ for binary datasets in the present simulation. In most conditions, NEST_poly_ was also more prone to underestimation than NEST_hybrid_. Notably, however, NEST_hybrid_ showed by far the most underestimations out of any method for binary datasets with *N* = 500 and varying asymmetric category probabilities (i.e., in the presence of difficulty factors). Therefore, while NEST_hybrid_ was more accurate in the presence of difficulty factors than its NEST_Pearson_ counterpart given that the latter was severely prone to overestimation, NEST_hybrid_ was ultimately less fit to handle difficulty factors in binary datasets in comparison to NEST_poly_.

**Four response categories**. Table 2 lists the proportions of outcomes for datasets with four response categories. NEST_Pearson_ was the most accurate method on average across all category probabilities with *N* = 500 (70.7% accurate, 15.0% underestimation, 14.2% overestimation) and with *N* = 100 (53.1% accurate, 43.7% underestimation, 3.1% overestimation).

**Table 2:** Outcomes for datasets with four response categories as a function of sample size and category probabilities

Method	Outcome	*N* = 100			*N* = 500		
		Symmetric	Invariant asymmetric	Varying asymmetric	Symmetric	Invariant asymmetric	Varying asymmetric
NEST_Pearson_	Overestimation	1.3	3.1	5.1	1.7	4.1	37.0
	Accurate	59.1	53.7	46.7	85.1	80.7	46.2
	Underestimation	39.7	43.2	48.2	13.2	15.2	16.8
	Undefined	0	0	0	0	0	0
NEST_poly_	Overestimation	0	0	0.1	0	0	0
	Accurate	25.6	30.0	29.4	45.1	70.4	45.2
	Underestimation	74.4	70.0	70.5	54.9	29.6	54.8
	Undefined	0	0	0	0	0	0
NEST_hybrid_	Overestimation	1.0	1.2	3.2	2.3	2.7	2.0
	Accurate	41.9	32.4	18.2	77.2	68.3	41.9
	Underestimation	57.1	66.4	78.5	20.5	29.0	56.1
	Undefined	0	0	0	0	0	0
PA_poly_	Overestimation	5.8	7.0	8.3	0	0.4	0.2
	Accurate	48.9	46.3	46.2	70.4	68.1	68.6
	Underestimation	45.3	46.7	45.5	29.6	31.5	31.2
	Undefined	0	0	0	0	0	0

Percentage of outcomes in all simulated datasets with four response categories.

Compared to binary datasets, NEST_Pearson_ and PA_poly_ improved (NEST_Pearson_: 41.6% accurate for all binary datasets, 61.9% accurate for all datasets with four categories; PA_poly_: 45.6% accurate for all binary datasets, 58.1% accurate for all datasets with four categories) while NEST_hybrid_ remained constant (46.6% accurate for all binary datasets, again 46.6% for all datasets with four categories) and NEST_poly_ became less accurate (47.8% accurate for all binary datasets, 41.0% accurate for all datasets with four categories). In summary, NEST_poly_ was the least accurate method for datasets with four categories.

The effects of sample size and category probabilities evident in Table 2 are similar to the effects of sample size and category probabilities from binary datasets in that methods mostly benefitted from increased sample size and symmetric category probabilities. An exception is that NEST_poly_ achieved its highest accuracy with *N* = 500 and invariant asymmetric category probabilities and was most likely to underestimate the number of factors with symmetric category probabilities. The low proportions of overestimations and the high proportions of underestimations by NEST_poly_ for four categories suggest that the inaccuracy of NEST_poly_ can be attributed to a severe lack of power. The inflation of the type 1 error rate in NEST_Pearson_ substantially exceeded its normative type 1 error rate of 5% only with *N* = 500 and varying asymmetric category probabilities. Crucially, as with binary datasets, results indicate that NEST_Pearson_ was more prone to overestimation with *N* = 500 than with *N* = 100 – particularly with varying asymmetric category probabilities. NEST_hybrid_ showed no inflated type 1 error rate for asymmetric category probabilities. However, NEST_hybrid_ notably showed frequent underestimation with varying asymmetric category probabilities unlike any other method, again hinting at a particularly strong decrement in power for NEST_hybrid_ in the presence of difficulty factors. In the end, no NEST variant outperformed PA_poly_ for datasets with four categories with varying asymmetric category probabilities.

#### Pilot simulation

All results for ordinal datasets can be put into perspective by comparing them to the performance of NEST_Pearson_ for continuous datasets in the pilot simulation. For continuous datasets, NEST_Pearson_ performed better with *N* = 500 (88.8% accurate, 10.0% underestimation, 1.2% overestimation) than with *N* = 100 (63.1% accurate, 35.0% underestimation, 1.9% overestimation).

The proportions of outcomes in Table 1 indicate that all methods performed worse for binary datasets than NEST_Pearson_ for continuous datasets. For binary datasets, NEST_Pearson_, NEST_hybrid_, and NEST_poly_ underestimated the number of factors more frequently across all category probabilities with *N* = 500 and *N* = 100. This indicates all NEST variants indeed suffered from decremented power compared to NEST_Pearson_ for continuous datasets, albeit NEST_poly_ and NEST_hybrid_ more so than NEST_Pearson_. However, overestimations by NEST_Pearson_ with asymmetric category probabilities further contributed to its decrement in accuracy for binary datasets compared to continuous datasets.

While NEST_Pearson_ improved for datasets with four categories, its performance was still worse than for continuous datasets. Compared to its performance for continuous datasets with *N* = 500 and *N* = 100, respectively, NEST_Pearson_ showed more underestimations across all category probabilities. With varying asymmetric category probabilities, overestimations by NEST_Pearson_ were also more frequent than for continuous variables. This pattern is similar to the results for binary datasets. It follows that, overall, reduced accuracy of NEST_Pearson_ for ordinal datasets compared to continuous datasets can be attributed to more frequent underestimation with all category probabilities and – simultaneously – more frequent overestimation with varying asymmetric category probabilities.

## Discussion

A key motivation to investigate NEST_Pearson_, NEST_poly_, and NEST_hybrid_ in a simulation was to test whether the anticipated problems for ordinal datasets would deteriorate their performance compared to the level of performance NEST has shown for continuous variables (Achim, 2017; Brandenburg & Papenberg, 2022). The results from the present simulated confirm the expected deterioration in that all three NEST variants performed worse for ordinal datasets than NEST_Pearson_ for continuous datasets. Concerning preference for one particular variant for application to ordinal datasets in applied research, implications in light of the current data are mixed. The general trends were that NEST_poly_ was superior for binary datasets with large sample sizes, that NEST_Pearson_ was superior for Likert-type datasets with homogeneous distributions of response categories among their variables, and that NEST_hybrid_ never emerged as superior over the other variants.

### Statistical power of NEST

There was reason to anticipate that the underestimation of model-implied correlations through sample Pearson correlations and the large standard error of sample polychoric correlations would result in deflated signal eigenvalues (see Fig. 1). The frequent underestimation of the optimal number of factors by NEST_Pearson_ and NEST_poly_ in the present simulation study implies that signal eigenvalues were indeed often too small to exceed their simulated sampling distribution when the null hypothesis of *k*-factor sufficiency was incorrect. Which NEST variant suffers from a stronger decrement in power was an open question that could not be answered without simulations. Interestingly, the stronger tendency toward underestimation by NEST_poly_ compared to NEST_Pearson_ for ordinal datasets suggests that polychoric correlations reduced the statistical power of NEST more than Pearson correlations despite the unbiasedness of polychoric correlations. At first, it may seem obvious to add the performance of NEST_hybrid_ to the discussion concerning power as a function of the type of correlation – after all, NEST_hybrid_ combines Pearson correlations and polychoric correlations and showed frequent underestimation of the number of factors itself. However, as will be discussed below, the reasons for underestimations of NEST_hybrid_ are best linked to its account for difficulty factors rather than properties of Pearson correlations and polychoric correlations.

Following reports that asymmetry in category probabilities increases bias of Pearson correlations and standard error of polychoric correlations (Lubbe, 2019), it was anticipated that the power of NEST_Pearson_ and NEST_poly_ depends on category probabilities. NEST_Pearson_ and NEST_poly_ mostly underestimated the number of factors more frequently with invariant or varying asymmetric category probabilities than with symmetric category probabilities. This trend is in line with reports from Lubbe (2019).

Inconsistent with this trend, NEST_Pearson_ underestimated the number of factors more often for binary datasets with *N* = 100 and symmetric category probabilities than with invariant asymmetric category probabilities. Also inconsistent with this trend, NEST_poly_ underestimated the number of factors for datasets with four categories most frequently with symmetric category probabilities. The observation of increased bias of Pearson correlations and standard error of polychoric correlations with asymmetric category probabilities, which can also be seen in Fig. 1, does not predict these notable underestimations with symmetric category probabilities. In total, this indicates that the dependence of NEST variants on the number of response categories and category probabilities is more complex than anticipated and requires further research. Still, in general, the present results add support to the notion that asymmetric category probabilities in ordinal datasets obstruct factor retention.

### Difficulty factors

Another problem with NEST_Pearson_ related to category probabilities was its alarming tendency to overestimate the number of factors for datasets with varying asymmetric category probabilities. This result fits the premise that difficulty factors occur in datasets when the correlation of item pairs not only depends on common factors but also on the distribution of response categories (Garrido et al., 2013; Green et al., 2016; Lim & Jahng, 2019; Tran & Formann, 2009; Yang & Xia, 2015) and that the continuous surrogate datasets simulated in NEST_Pearson_ fail to account for category probabilities as a confounded source of correlation. Thus, the tendency toward overestimation by NEST_Pearson_ provides evidence that the original implementation – which was not designed for application to ordinal variables – indeed fails to safeguard against retention of difficulty factors.

The overestimations by NEST_Pearson_ in the presence of difficulty factors occurred more frequently with *N* = 500 than with *N* = 100. The reason for this was the sample Pearson correlations were less noisy with *N* = 500 than with *N* = 100. Tested eigenvalues corresponding to a difficulty factor more often exceeded their simulated sampling distribution that did not account for a difficulty factor since this difference was not attributed to noise.

Throughout the present work, it was assumed that difficulties do not add to the optimal number of factors and that their retention can be considered as overestimation, which is common practice in simulation studies on the number-of-factors problem in ordinal datasets (see Garrido et al., 2013; Lim & Jahng, 2019; Tran & Formann, 2009). This assumption may be challenged on the ground that difficulty factors in Pearson correlation matrices are no product of noise but are in fact necessary components to model observed correlations whenever item difficulty varies within a set of ordinal variables (Olsson, 1979b). However, given that difficulty factors do not reflect psychological constructs (Lim & Jahng, 2019; Lubbe, 2019; Tran & Formann, 2009), difficulty factors should be separated from substantive factors (i.e., factors that do reflect psychological constructs) in the interpretation of factor models that retain difficulty factors. Consequently, when the motivation to do exploratory factor analysis is to explore common constructs in an observed dataset, a method to determine the number of factors that is more likely to retain difficulty factors (e.g., NEST_Pearson_) than other methods but not more accurate with respect to substantive factors serves no benefit. NEST_Pearson_ is therefore not recommended for ordinal datasets with varying asymmetric category probabilities.

The adapted implementation NEST_hybrid_ was proposed to counteract the expected tendency toward overestimation of NEST_Pearson_ in the presence of difficulty factors. This was done by simulating ordinal surrogate datasets instead of continuous datasets, replicating the influence of difficulty factors in surrogate datasets by reproducing the observed category probabilities in the surrogate datasets. Overall, in the present simulation, NEST_hybrid_ unlike NEST_Pearson_ did not exceed 5% probability of overestimation of the number of factors, including datasets with varying asymmetric category probabilities. This verifies that NEST_hybrid_ was indeed successful to counteract the issue of overestimation by NEST_Pearson_ in the presence of difficulty factors.

On the other hand, it must be noted that NEST_hybrid_ showed the highest probability of underestimations of all methods in most conditions with varying asymmetric category probabilities (see Tables 1 and 2). This shows that NEST_hybrid_ lacked statistical power compared to NEST_Pearson_ and NEST_poly_ in the presence of difficulty factors. To understand the lack of power, recall that the ordinal surrogate datasets in NEST_hybrid_ reproduced observed category probabilities. Hence, the influence of difficulty factors was replicated in the surrogate datasets for the test of every eigenvalue from the analyzed sample correlation matrix, starting with the first eigenvalue at *k* = 0. Also, recall that difficulties are assumed to manifest in elevated noise eigenvalues that do not belong to the set of signal eigenvalues corresponding to factors from the population-model (Garrido et al., 2013; Lim & Jahng, 2019). When testing signal eigenvalues in the conditions with varying asymmetric category probabilities in present simulation, Pearson correlation matrices in NEST_hybrid_ accounted for difficulty factors and thus included the corresponding elevated noise eigenvalues. The Pearson correlation matrices for continuous surrogate datasets in NEST_Pearson_ did not include these elevated noise eigenvalues. Therefore, in the presence of difficulty factors, the simulated sampling distribution for tested signal eigenvalues included in NEST_hybrid_ consisted of greater eigenvalues than in NEST_Pearson_, thereby increasing the threshold for significance in NEST_hybrid_.

In the end, the account for difficulty factors in NEST_hybrid_ which protected against overestimation in their presence also reduced its power in the prior tests of signal eigenvalues. Overall, NEST_hybrid_ was less accurate on average than NEST_poly_ across all conditions with binary datasets, less accurate on average than NEST_Pearson_ across all conditions with four response categories, and its supposed theoretical advantage in the account for difficulty factors ultimately caused NEST_hybrid_ to underperform compared to NEST_poly_ or NEST_Pearson_ with varying asymmetric category probabilities. Therefore, NEST_hybrid_ cannot be recommended as the preferred implementation of NEST in light of the current data and is dropped from further discussion.

### Empirical example

Lubbe (2019) applied PA_poly_ to an empirical dataset with binary variables to test whether polychoric correlations and reproduced category probabilities in surrogate datasets prevent sensitivity to difficulty factors in PA. Here, their analysis was replicated with NEST_Pearson_, NEST_poly_, and NEST_hybrid_. The dataset is a sample (*N* = 150) of Bond’s Logical Operations Test, which includes 35 binary variables (Bond & Fox, 2007, as cited by Revelle, 2022a, 2022b); it is available in the R package *psychTools* (Version 2.2.5; Revelle, 2022a, 2022b; retrievable as *psychTools::blot*) as a toy dataset in the context of item response theory. As such, the items can be assumed to reflect a single common factor. The items’ mean difficulty – quantified as the proportion of correct answers per item – is 0.75 (*SD* = 0.13) with two item difficulties below 0.50 (i.e., 0.36, 0.49). Hence, the dataset can be considered in between the category probability levels ‘invariant asymmetric’ and ‘varying asymmetric’ of the present simulation. NEST_poly_ and NEST_hybrid_ suggested one factor while NEST_Pearson_ suggested three. This example is in line the finding from the present simulation that NEST_Pearson_ is a more liberal method to determine the number of factors compared to other methods for ordinal datasets with asymmetric category probabilities, which should be kept in mind in empirical applications.

### Recommendations

One goal of the research reported here was to compare NEST_Pearson_, NEST_poly_, and NEST_hybrid_ to explore potential guidelines about which variant should be preferred for ordinal datasets. The present simulation was deliberately designed to simulate situations in which the anticipated problems of these methods should be easily observed, facilitating an assessment of their relative differences. More importantly, however, the low accuracies obvious from Tables 1 and 2 indicate that neither method achieved satisfactory performance under several conditions in the present simulation and that preference should be adopted with respect to features of the dataset at hand. The following sections summarize the present results with the intent to point out explicitly which implementation of NEST, if any, can be recommended for applied research depending on features of the data. As mentioned, since NEST_hybrid_ was always outperformed by either NEST_Pearson_ or NEST_poly_, NEST_hybrid_ is not considered in this section.

Note that recommendations of NEST_Pearson_ and NEST_poly_ only apply to the choice between Pearson correlations and polychoric correlations in NEST. Given that NEST only serves the determination of the optimal number of factors, which is considered the same for sample correlation matrices with Pearson correlation and polychoric correlations from the same population, preference for either type of correlation in NEST does not imply preference for the same type in the subsequent parameter estimation for a factor model with the respective number of factors (see Flora & Curran, 2004).

With *N* = 100, no method reached 50% accuracy for binary datasets and no method reached 60% accuracy for datasets with four response categories. Based on the present results, *N* = 500 is recommended as the minimum sample size to tackle the number-of-factors problem in ordinal datasets with four or fewer response categories. The levels of sample size (100; 500) in the present simulation do not justify recommendation of a lower minimum sample size.

With *N* = 500, in relative terms, NEST_poly_ outperformed NEST_Pearson_ for binary datasets: NEST_poly_ was more accurate than NEST_Pearson_ and it remained within its normative type 1 error rate while the type 1 error rate of NEST_Pearson_ was strongly inflated with asymmetric category probabilities. Hence, NEST_poly_ seems to be the preferable NEST variant for binary datasets with *N* = 500. However, given that NEST_Pearson_ was substantially more accurate than NEST_poly_ for datasets with four categories, the benefits of polychoric correlations to NEST outweigh their costs only for binary datasets.

For ordinal datasets with more than two response categories, NEST_Pearson_ seems to be the preferred NEST variant. As the bias of Pearson correlations decreases with an increasing number of response categories (see Fig. 1; Green et al., 2016), it can be assumed that NEST_Pearson_ further improves with more than four response categories. This assumption was verified in an additional simulation that included the same conditions as the simulation reported above but with five response categories per variable (category probabilities for symmetric, invariant asymmetric, and varying asymmetric distributions were adopted from Goretzko & Bühner, 2022). The results of this additional simulation are reported in Appendix B. Consistent with the present results on four response categories, NEST_Pearson_ overall outperformed NEST_poly_, NEST_hybrid_, and PA_poly_ for datasets with five response categories, and was outperformed only by PA_poly_ with *N* = 500 and varying asymmetric category probabilities due to frequent overestimation in the presence of difficulty factors. Since NEST_Pearson_ failed to outperform PA_poly_ for datasets with more than two response categories and varying asymmetric category probabilities, a recommendation of NEST_Pearson_ for ordinal datasets with more than two response categories should be limited to homogeneous item difficulties in light of its sensitivity to difficulty factors. For datasets with more than two categories, more research is required to develop a method that is as robust against varying item difficulties as PA_poly_ while also improving on theorical flaws of PA (see Braeken & van Assen, 2017; Green et al., 2012; Ruscio & Roche, 2012; Saccenti & Timmerman, 2017; Turner, 1998).

The observed errors by all methods investigated in the present simulation highlight the need for guidelines to qualify a suggested number of factors as optimal in applied research where – unlike in simulations – no ground-truth typically exists. Such guidelines are particularly important for ordinal datasets given that ordinal datasets promoted underestimations *and* overestimations by NEST, depending on the implementation. In general, the optimal solution to the number-of-factors problem does not miss factors of substantive importance and does not retain factors than suit no sound interpretation (Braeken & van Assen, 2017; Preacher et al., 2013).

Underestimations by NEST indicate that failure to reject the null hypothesis of *k*-factor sufficiency for a tested eigenvalue does not imply that the eigenvalue corresponds to a negligible factor. A guideline to avoid missing a factor with NEST is to employ large samples to increase its statistical power, which is supported by the present simulation (*N* ≥ 500 for ordinal datasets). When large samples are infeasible, the number of factors in exploratory factor analysis may be increased as long as the added factors provide contributions to the factor model that are deemed substantial according to the interpretation of model parameters in light of domain-specific theory.

What is more, overestimations by NEST indicate that statistical significance of an eigenvalue in NEST does not imply that the eigenvalue corresponds to substantive contribution by a distinct factor (Brandenburg & Papenberg, 2022). Known alternative explanations for significance in NEST are mere sampling variance (i.e., a type 1 error), the unaccounted presence of a difficulty factor, or cumulative contributions of minor sources of correlations that do not correspond to a factor (Achim, 2021; Auerswald & Moshagen, 2019; Cosemans et al., 2022; Lim & Jahng, 2019). These alternative explanations may also lead one to consider numbers of factors below the solution of NEST when not all factors accepted by NEST are interpretable post rotation (see Fabrigar et al., 1999).

A strategy to avoid relying on potentially erroneous solutions suggested in multiple recent publications is to consider solutions of different methods (Auerswald & Moshagen, 2019; Goretzko et al., 2021; Preacher et al., 2013). Combining different solutions requires that the relative performance of the respective methods is well-understood (Li et al., 2020) in order to interpret conflicting solutions of different methods. In the present simulation, heterogeneous item difficulties in ordinal datasets caused frequent overestimations by NEST_Pearson_ and frequent underestimations by NEST_poly_. Following this observation, it was tested whether a combination rule of NEST_Pearson_ and NEST_poly_ would improve accuracy with heterogeneous item difficulties by treating the solution by NEST_poly_ as the lower bound for the number of factors and the solution by NEST_Pearson_ as the upper bound. To this end, only datasets from the present simulation with varying asymmetric category probabilities and identified solutions (i.e., not unidentified solutions as in Tables 1 and 2) by NEST_Pearson_ and NEST_poly_ were considered. The boundaries covered the true number of factors in 29.4% of binary datasets with *N* = 100, in 62.5% of binary datasets with *N* = 500, in 51.7% of datasets with four categories with *N* = 100, and in 83.3% of datasets with four categories and *N* = 500. Compared to the accuracies listed in Tables 1 and 2, the boundaries covered the true number of factors more often than it had been hit by NEST_Pearson_ and NEST_poly_ individually. When this combination rule is applied to the 35 binary variables from the empirical example of Bond’s Logical Operations Test (Bond & Fox, 2007, as cited by Revelle, 2022a, 2022b), NEST_poly_ indicates one factor as the lower bound and NEST_Pearson_ indicates 3 factors as the upper bound. A thorough examination of the factor loadings patters in the three factor models – with one, two, and three factors respectively – could then guide toward the preferred solution based on their respective theoretical interpretability.

An obvious problem with this combination rule is that varying asymmetric category probabilities not only promoted overestimations by NEST_Pearson_ but also underestimations. Hence, NEST_Pearson_ should not be expected to provide a reliable upper bound for the optimal number of factors. Still, given that factor retention for ordinal variables with heterogeneous item difficulties remains challenging for NEST and PA alike, this combination rule may be a useful heuristic that exploits shortcomings of the individual methods. This combination rule hence offers potential prospect for further research.

The current comparison of NEST_Pearson_ and NEST_poly_ also has implications for simulation studies aimed at investigating methods to determine the number of factors similar to the present study. For instance, Lim and Jahng (2019) compared traditional PA to RPA – which is highly similar to NEST – in a simulation that included datasets with four response categories. Their tested implementation of RPA computed polychoric correlations for the analyzed dataset and for categorized surrogate datasets, similar to the present implementation of NEST_poly_. In light of the present simulation, which limits benefits of polychoric correlations for NEST to binary datasets, Lim and Jahng likely tested a suboptimal implementation of RPA. The present results suggest that further simulation studies which include NEST or RPA as well as ordinal datasets should include a variant that computes Pearson correlations even for ordinal datasets.

### Limitations

A limitation of the present work is that the simulation was designed with a restricted set of conditions that did not target optimal conditions for NEST and PA. Given that both NEST and PA are eigenvalue-based methods, their performance can be expected to improve for ordinal datasets when factors explain more variance across all variables. For Pearson correlation matrices and polychoric correlation matrices, signal eigenvalues are larger, for instance, in datasets with more variables per factor (Auerswald & Moshagen, 2019). However, it requires further simulations to test whether there are conditions that nullify the identified problems with NEST_Pearson_, NEST_poly_, and NEST_hybrid_ for ordinal datasets.

The present simulation may be considered idealistic in that it did not account for cross-loadings of simulated variables on multiple factors (Brandenburg & Papenberg, 2022; Li et al., 2020). Given that the number-of-factors problem implies that it is unknown how many factors inform the variables, it should not be assumed in exploratory factor analysis that variables are informed by one common factor each (Achim, 2020). Model-implied correlation matrices of factor models with substantial cross-loadings in the majority of variables can yield lower signal eigenvalues (excluding the largest signal eigenvalue) than correlation matrices of factor models without cross-loadings, mimicking the effect of inter-factor correlations (see Brandenburg & Papenberg, 2022, for a detailed explanation of this effect). The effects of inter-factor correlations and cross-loadings may add up and thus provide more challenging conditions than the ones included in the present simulation. The results from the present work already indicate that, even when cross-loadings can be assumed absent, there are conditions that challenge the power of NEST to such a high extent that all variants (as well as PA) failed to achieve satisfactory accuracy (i.e., binary datasets with small sample sizes). Conditions in which the reduction of signal eigenvalues due to inter-factor correlations and cross-loadings add up would further increase the risk of underestimation of the optimal number of factors. Therefore, future research targeting ordinal datasets should assess to what extent ordinal variables with cross-loadings deteriorate the performance of NEST when neither inter-factor correlations nor cross-loadings can be assumed absent.

Furthermore, the present work only accounts for ordinal datasets in which all variables have the same number of response categories. The present results do not generalize to datasets with mixed scales. Further research is needed to address optimal estimation of correlation in NEST for mixed datasets.

Another technical limitation concerns the computation of the *k*-factor models in NEST. In all tested NEST implementations from the current work, iterative reduction of the sample correlation matrix was used in accordance to Achim (2017). Achim (2017) provides an in-depth explanation of this method to compute the *k*-factor models and a comparison to other approaches in which the iterative reduction resulted in the highest accuracy of NEST. However, these results only apply to *k*-factor models of Pearson correlation matrices for continuous variables. It requires additional dedicated research to investigate if the iterative reduction that was used in all present implementations of NEST is optimal when *k*-factor models are computed for polychoric correlation matrices or if other approaches would yield better performance of NEST.

Finally, another limitation concerns the assumption of normally distributed latent variables by the maximum-likelihood estimator of polychoric correlations (Olsson, 1979a). Whenever polychoric correlations were computed (i.e., Fig. 1, NEST_poly_, NEST_hybrid_, PA_poly_), ordinal variables had been simulated by transforming normally distributed variables using predetermined thresholds (Yang & Xia, 2015). Therefore, the distributional assumptions of the estimator of polychoric correlations were never violated. However, assuming that ordinal variables are discrete indicators specifically of normally distributed continuous variables may not hold in empirical applications. As an example, Jin and Yang-Wallentin (2017) pointed out that income as an indicator of socio-economic status may not be normally distributed due to its natural lower bound but still may be measured in ordered categories. In the present implementation of NEST_poly_, the simulated ordinal surrogate datasets always met distributional assumptions of polychoric correlations by design. This raises the question if NEST_poly_ does provide adequate sampling distributions of tested eigenvalues when the analyzed dataset unilaterally violates distributional assumptions. The present work provides no indication of the performance of NEST_poly_, NEST_hybrid_ or PA_poly_ when distributional assumptions of polychoric correlations are violated. Therefore, further research is required to test the benefits and costs of polychoric correlations for factor retention in the presence of violated distributional assumptions.

### Conclusion

All in all, the present work shows that the performance of NEST for ordinal variables depends on properties of computed correlations (i.e., bias, standard error). The present simulation provides evidence that polychoric correlations for analyzed datasets and ordinal surrogate datasets benefit the performance of NEST in retaining the optimal number of factors for binary datasets. However, the tested implementation of NEST using polychoric correlations required large samples to achieve satisfactory performance (*N* ≥ 500) and the benefits of polychoric correlations did not extend to ordinal datasets with more than two response categories per variable. For datasets with four response categories, the problems of polychoric correlations were more severe than the problems of Pearson correlations. In general, the present simulation suggests that factor retention is more error-prone for ordinal datasets than for continuous datasets. More research addressing ordinal variables is required to investigate which method is optimal under which condition and how potentially suboptimal solutions are handled in empirical applications of exploratory factor analysis.

## Data Availability

The raw results of the current simulation study are available in the Open Science Foundation repository: https://osf.io/wb2ys/.

## Appendix A

### Implementation details

All simulations and analyses in the present work were conducted in the statistical programming environment R (Version 4.1.0; R Core Team, 2021). Ordinal datasets were simulated by transforming normally distributed continuous variables into ordered categories. To simulate $p$ variables in accordance to a factor model, random variables were sampled from the multivariate normal distribution 𝒩(${0}_{p}$, $\Sigma$), with ${0}_{p}$ denoting the vector of *p* variable means (all set to 0) and $\Sigma$ denoting the $p\times p$ model-implied correlation matrix using the *rmvnorm* function from the R package *mvtnorm* (Version 1.1–3; Genz et al., 2021).

As for the computation of polychoric correlations, two different functions were used for binary datasets and datasets with four response categories. For binary datasets, polychoric correlations – which could also be referred to as tetrachoric correlations in the binary case – were computed with the *tetrachoric2* function from the R package *sirt* (Version 3.12–66; Robitzsch, 2022). For datasets with four categories, polychoric correlations were computed with the *polychoric* function from the R package *psych* (Version 2.2.5; Revelle, 2022a, 2022b). The psych implementation would have worked also for binary datasets, but the sirt implementation was preferred because it was significantly faster than the psych implementation, which greatly facilitates applications of NEST_poly_ in large-scale simulations.

A common problem with the estimation of polychoric correlations are nonpositive definite correlation matrices (Garrido et al., 2013; Green et al., 2016; Timmerman & Lorenzo-Seva, 2011; Weng & Cheng, 2017). This implies that some eigenvalues of the sample correlation matrix may be negative, which contradicts their interpretation in the present context as indicators of variance explained by factors. The default setting to handle nonpositive definite correlation matrices for polychoric correlations in sirt and psych – as of their respective versions in the present work – is to apply the smoothing procedure from the psych package, which rescales estimated polychoric correlation matrices in a way that all eigenvalues are nonnegative. The default smoothing procedure was retained in all estimations of polychoric correlations in the present work.

## Appendix B

### Additional simulation

An additional simulation was carried out to verify further that NEST_Pearson_ outperforms NEST_poly_, NEST_hybrid_, and PA_poly_ for ordinal datasets with more than two response categories per variable. The main simulation from the current work only included ordinal variables with four response categories in this regard. Goretzko and Bühner (2022) conducted similar simulations with ordinal variables and proposed a method to simulate datasets with five response categories. Like the method from Yang and Xia (2015) that was used in the present work, the method from Goretzko and Bühner (2022) used intervals under the standard normal distribution corresponding to predetermined threshold values to manipulate expected category probabilities. Their method was suitable for the present work as it provides threshold values for symmetric ({– 0.84, – 0.25, 0.25, 0.84}) and asymmetric ({– 0.08, 0.25, 0.62, 1.11}) category probabilities. For the additional simulation here, these thresholds were used to simulate symmetric, invariant asymmetric (i.e., applying the according thresholds for every variable) and varying asymmetric (i.e., applying the asymmetric thresholds, but in sign-reversed form for every second variable) category probabilities just as in the main simulation from the present work. In addition, the additional simulation also included the same manipulations of the true number of factors (2, 4), the number of variables per factor (4, 7), the distribution of loading parameters (*𝒰*(0.40, 0.50), *𝒰*(0.70, 0.80)), the inter-factor correlation parameters (0.20, 0.70), the sample size of datasets (100, 500) as the main simulation in a fully crossed design. In total, 100 datasets were simulated per condition, yielding 9600 datasets with five response categories per variable.

Table 3 lists the performances of all methods in the same fashion as above. Averaged across all simulated datasets, NESTP_earson_ was the most accurate method (66.4%), followed by PA_poly_ (58.3%), NEST_hybrid_ (49.1%), and finally NEST_poly_ (41.9%).

**Table 3:** Outcomes for datasets with five response categories as a function of sample size and category probabilities

Method	Outcome	*N* = 100			*N* = 500		
		Symmetric	Invariant asymmetric	Varying asymmetric	Symmetric	Invariant asymmetric	Varying asymmetric
NEST_Pearson_	Overestimation	1.7	3.4	2.2	1.8	3.1	17.4
	Accurate	58.6	54.1	52.0	85.4	82.4	66.0
	Underestimation	39.8	42.4	45.8	12.8	14.4	16.6
	Undefined	0	0	0	0	0	0
NEST_poly_	Overestimation	0	0	0	0	0	0
	Accurate	26.7	30.4	28.9	46.5	69.8	49.0
	Underestimation	73.3	69.6	71.1	53.5	30.2	51.0
	Undefined	0	0	0	0	0	0
NEST_hybrid_	Overestimation	0	0	0.1	0	0.1	0
	Accurate	30.9	37.2	30.5	66.9	78.7	50.2
	Underestimation	69.1	62.7	69.4	33.1	21.2	49.8
	Undefined	0	0	0	0	0	0
PA_poly_	Overestimation	5.8	7.7	7.9	0	0.4	0.2
	Accurate	49.5	45.2	46.6	69.6	69.8	69.0
	Underestimation	44.7	47.1	45.6	30.4	29.9	30.8
	Undefined	0	0	0	0	0	0

Percentage of outcomes in all simulated datasets with five response categories.

## Footnotes

1 The computation of the *k*-factor models does not involve any form of factor rotation. The goal in NEST is to compute a model with *k* latent dimensions that explain as much variance in sum as *k* rotated factors would. This does not require rotation of the *k* dimensions since rotation does not change the sum of variance that is explained by the model. Nor does rotation change the eigenvalues of the model-implied correlation matrix or – by extension – the eigenvalues from sample correlation matrices under the model. The models for the different values of *k* that are computed in NEST are hence referred to as *k*-factor models to highlight their goal of carrying over the total variance explained by the first *k* factors into the surrogate datasets.

2 This explanation for difficulty factors was adopted from an anonymous reviewer to a previous submission of this work.

3 Polychoric correlations for binary variables may also be referred to as tetrachoric correlations. Here, the term polychoric correlations is used for consistency with the naming for variables with four response categories.

4 An implementation of NEST that used polychoric correlations for analyzed datasets and Pearson correlations for continuous surrogate datasets was tested in an unpublished simulation. The results confirmed that this implementation vastly overestimates the number of factors, disqualifying it from further consideration.

## Code availability

The source code to all reported analyses, all investigated methods, and all reported simulations (including the ones to generate the displayed figures) are available in the Open Science Foundation repository: https://osf.io/wb2ys/.
